# A Tissue Digestion Protocol for Measuring *Sarcoptes scabiei* (Astigmata: Sarcoptidae) Density in Skin Biopsies

**DOI:** 10.1093/jisesa/ieaa105

**Published:** 2020-11-02

**Authors:** Hannah S Tiffin, Robert Cockerill, Justin D Brown, Erika T Machtinger

**Affiliations:** 1 Department of Entomology, 4 Chemical Ecology Laboratory, Pennsylvania State University, University Park, PA; 2 Department of Veterinary and Biomedical Sciences, 4 Chemical Ecology Laboratory, Pennsylvania State University, University Park, PA; 3 Department of Veterinary and Biomedical Sciences, 110 Research Unit A, Pennsylvania State University, University Park, PA

**Keywords:** mange, *Sarcoptes scabiei*, tissue digestion, wildlife, canid

## Abstract

Sarcoptic mange is a parasitic skin disease caused by the burrowing mite *Sarcoptes scabiei* that affects a diversity of mammals, including humans, worldwide. In North America, the most commonly affected wildlife includes wild canids, such as coyotes and red foxes, and more recently American black bears in the Mid-Atlantic and Northeast United States. Currently, surveillance for sarcoptic mange in wildlife is syndromic, relying on detection of clinical signs and lesions, such as alopecia and crusting of skin. When possible, skin scrapes are used to identify the causative mite. While skin scrapes are a valuable diagnostic tool to identify mites, this approach has significant limitations when used for quantification of mite burden. To further investigate mite burden in cases of sarcoptic mange, 6-mm punch biopsies were collected from affected skin of red foxes (*Vulpes vulpes* Linnaeus [Carnivora: Canidae]), a species historically affected by sarcoptic mange, frequently with high mite burdens and severe skin disease, and validated on skin tissue from mange-affected American black bears (*Ursus americanus* Pallas [Carnivora: Ursidae]) and coyotes (*Canis latrans* Say [Carnivora: Canidae]). Biopsies were digested by incubating the tissue in potassium hydroxide (KOH) at 55°C. The greatest tissue clearance and lowest mite degradation resulted after 12 h of tissue digestion. The purpose of this manuscript is to describe a methodology for host tissue digestion and mite quantification in cases of sarcoptic mange. This method will provide a valuable surveillance and research tool to better understand sarcoptic mange in wild and domestic animals, with applications to a diversity of other ectoparasitic diseases.

Sarcoptic mange is a parasitic skin disease, caused by the microscopic mite *Sarcoptes scabiei* (De Geer [Astigmata: Sarcoptidae]), that affects over 100 different species of mammals worldwide, including humans (Bornstein et al. 2001, Pence and Ueckermann 2002, Simpson et al. 2016). Disease associated with *Sarcoptes scabiei* is due to a combination of direct damage to the skin and adnexal structures from the mite burrowing through the epidermis, and the host’s hypersensitivity reaction to the foreign parasitic material (i.e., mites, eggs, and feces). Collectively, these result in clinical signs such as varying alopecia, thickened and crusted skin, dermatitis, and darkened pigmentation of the skin (Pence and Ueckermann 2002), as well as predisposing the affected animal to secondary infections (Simpson et al. 2016, Peltier et al. 2018, Niedringhaus et al. 2019a). In severely affected animals, severe skin disease can result in listlessness, emaciation, dehydration, and in some cases death (Bornstein et al. 2001, Pence and Ueckermann 2002).

Historically, sarcoptic mange has caused epizootics among populations of wild canids in North America, such as red foxes (*Vulpes vulpes* Linnaeus [Carnivora: Canidae]) and coyotes (*Canis latrans* Say [Carnivora: Canidae]) (Pence et al. 1983, Pence and Ueckermann 2002, Niedringhaus et al. 2019a). Black bears (*Ursus americanus* Pallas [Carnivora: Ursidae]) were only rarely reportedly with sarcoptic mange, but since the 1990s reports of black bears with mange have been increasing in Pennsylvania and cases have been increasingly reported from surrounding states (Niedringhaus et al. 2019b).

Host response to parasitism by *S. scabiei* can vary dramatically between individuals and across species. Individuals may have low mite densities but severe clinical signs and lesions, while other individuals may have high mite densities but show mild to moderate clinical signs and lesions. The individual host response to infestation creates discrepancies in observed clinical manifestation and genuine severity of infestation (Bornstein et al. 2001, Pence and Ueckermann 2002, Arlian and Morgan 2017, Peltier et al. 2018). Additionally, infestation with *S. scabiei* can lead to different host hypersensitivity responses. For example, experimentally infested red foxes (Little et al. 1998) and wild coyotes (Pence et al. 1983) developed a predominantly type I (immediate) hypersensitivity response to infestation while other animals such as domestic dogs and pigs demonstrated both types I and IV (immediate and delayed) hypersensitivity responses (Pence and Ueckermann 2002). Combined, the differences in mite burden, host response, and clinical signs and associated lesions present unique challenges to diagnosis of animals afflicted with sarcoptic mange.

Sarcoptic mange is diagnosed by confirming the presence of *S. scabiei* mites, through morphologic or molecular identification, in skin with characteristic lesions. Detection of sarcoptic mange in wildlife typically relies on syndromic surveillance (i.e., identification of outwardly apparent clinical signs). If the animal can be handled, skin scrapes are collected from affected skin and mites are identified microscopically by morphology. While skin scrapes are a valuable and simple tool that can be used to identify mites, this approach is inaccurate for determining mite density due to the inability to collect scrapes in a standardized manner, both by depth and by the amount of skin lesion sampled. Additionally, it is difficult to view mites still burrowed in mite tunnels when using skin scrapes (Arlian and Morgan 2017, Peltier et al. 2018).

To aid in diagnosis, slides made from skin scrapings can be heated to promote movement of the mites out of the host’s skin tissue to better enable confirmation of mites (Bornstein et al. 2001, Alasaad et al. 2009, Castro et al. 2018). However, this does not enable diagnosticians and researchers to accurately count the number of mites present in skin scrapings and biopsies as not all mites migrate out of the skin. Thus, the use of this method still does not accurately address questions of mite density in a sample (Alasaad et al. 2009, Castro et al. 2018).

Another technique used is the digestion of host tissue in a potassium hydroxide (KOH) solution. This procedure dissolves keratin, proteins, and lipids, but structures containing chitin remain intact (Afshar et al. 2018), thereby increasing the visibility of chitinous *S. scabiei* exoskeletons using microscopy (Bornstein et al. 2001). While this common method is used for mite identification from skin scrapes or biopsies or in studies on *S. scabiei* infestations, there are few published resources available on methodology. A study by Davis and Moon (1990) provides procedural information regarding materials used and the digestion time employed; however, the filter referenced in their procedure is no longer available and the manufacturer no longer has records dating to this time and is thus unable to recommend a similar currently available item.

Research efforts to better understand *S. scabiei* infestation in wildlife are currently hindered by lack of diagnostics to quantify mite burden. The goal of this study was to evaluate a standardized approach to determining mite burden in animals with sarcoptic mange. This protocol was developed to efficiently determine the number of mites present in mange-affected skin tissue. The protocol was developed by using tissue from red foxes as this is a species commonly affected by sarcoptic mange, frequently with high mite burdens and severe skin disease (Pence and Ueckermann 2002, Little et al. 1998). The protocol was then validated on two additional species affected by sarcoptic mange, the American black bear and the eastern coyote. This protocol can be used for confirmatory diagnosis from skin scrapings and biopsies or for determining infestation levels according to mite densities for research or treatment purposes. This methodology can be employed by diagnosticians, clinicians, parasitologists, and researchers investigating *S. scabiei* infestation, other mite infestations, and for the digestion of tissues associated with other diseases. Additionally, the KOH digestion method has been used in other applications as well, in fields as divergent as microplastic pollution in marine organisms (Thiele et al. 2019).

## Materials

- 6-mm disposable biopsy punch, sterile (Integra Miltex, York, PA)
- Square petri dish, electron microscopy sciences, 100 × 15 mm dish (VWR International, Radnor, PA)
- Potassium hydroxide pellets (Fisher Chemical, Ottawa, ON, Canada)
- Hot plate (Thermo Fisher Scientific Fisherbrand Isotemp stirring hotplate, Shanghai, China)
  - Note: Fisher Scientific Representatives do not recommend using plastic or polystyrene petri dishes with prolonged heat exposure above 60ºC
- Stereoscope, 50× magnification or higher (8X-50X Track Stand LED Light Stereo Zoom Parfocal Trinocular TouchPad Microscope, AmScope, Los Angeles, CA)

## Procedure

### Sample Collection

All of the skin biopsies included in this study were collected from animals euthanized due to severe signs of mange, such as thickened crusting skin, lesions, and emaciation. Animals used in development of this protocol were submitted as part of a larger study on mange between the dates of 18 February 2019 and 26 June 2020, with samples typically collected the day the animal was euthanized but always within 2 d of death. All animal tissue used for validating the timing trials in this protocol were from animals confirmed to have sarcoptic mange through identification of *S. scabiei* on skin scrapes. Five animals with moderate to high mite burdens were specifically selected for inclusion in this study to observe trends in mite persistence throughout the timing trials. Two additional animals with low mite burdens were included in this study to evaluate the protocol’s utility for skin tissue with low mite densities. Full-thickness skin samples were preferentially collected from areas of skin that were most severely affected, as evidenced by alopecia, skin crusting, thickened skin, and when possible, locations where mites were observed in initial skin scrapes. Samples were placed in individual sealable plastic bags and stored at −20°C until the digestion trials were performed. All work and sample collection was conducted under the authorization of the Pennsylvania Game Commission (PGC) permit # 42115 and Pennsylvania State University (PSU) Institutional Animal Care and Use Committee protocol (IACUC #47978).

### Digestion Timing Trials: Protocol Validation

**Fig. 1.**
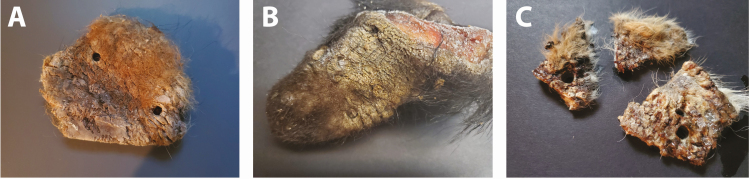
Photograph of characteristic sarcoptic mange lesions, and from which high mite burden was identified using the KOH digestion procedure described in this paper. Biopsies were removed from these lesions for use in the high mite burden timing trials, with tissue from (a) a red fox, (b) a black bear, and (c) a coyote, all with severe lesions, alopecia, and skin crusting present.

Twenty biopsies from five different animals were evaluated at six specified time points—4, 8, 12, 16, 24, and 48 h post-digestion. Timing trials were initially evaluated on eight biopsies collected from one red fox. The eight biopsies represented individual replicates that were evaluated for tissue digestion and mite quantification over these six time points. To validate this procedure on different individuals and different species, four additional animals with three biopsies from each individual were evaluated. All trials from these animals were evaluated over these same six time points as for the original red fox. In total, this procedure was validated on tissue collected from two red foxes, two black bears, and one coyote affected with severe sarcoptic mange. Using the same methodology, two additional animals with low mite burdens were evaluated to determine the utility of this methodology for animals with low mite densities. These trials consisted of two biopsies from one black bear and four biopsies from one coyote. These trials were in addition to the timing trials conducted on the five animals with high mite burdens. Each biopsy was digested in a separate individual petri dish and evaluated at these six time points. For each digestion, one 6-mm biopsy was collected (6-mm sterile disposable biopsy punch, Integra Miltex, York, PA) from an area with thickened, crusted lesions (Fig. 1).

### KOH Digestion Procedure

Each skin biopsy was placed in an individual polystyrene square-gridded 100 × 15 mm Petri dish (VWR International, Radnor, PA) and filled with approximately 50–60 ml of 10% KOH solution (KOH pellets, Fisher Chemical, Ottawa, ON, Canada) until the biopsy was submerged in the solution. Petri dishes were covered with a lid and placed on a hot plate at 55°C. Mites were counted at 4, 8, 12, 16, 24, and 48 h post-digestion.

### Mite Density Counts

At each time point, the petri dish was removed from the hot plate and immediately placed under a stereomicroscope and examined under 50× magnification (AmScope, Los Angeles, CA). The total number of mites identified per plate and presence or absence of each life-stage were recorded (i.e., adult female, adult male, juvenile, eggs). Two options were determined to improve the ability to identify and count the mites in the solution. The first option was to add a Double Stain (BioQuip Products Inc., Rancho Dominguez, CA) to the digestion solution to improve mite contrast with a light-colored (white or clear) stereoscope stage plate. The second option used a dark stage plate to increase contrast of mites in the solution without the use of the Double Stain, and is recommended over use of the Double Stain as the mites were more clearly visible with the dark stage plate. It is the authors’ opinion that the latter option was more effective and it is what was used for the trials reported herein.

## Results

**Fig. 2.**
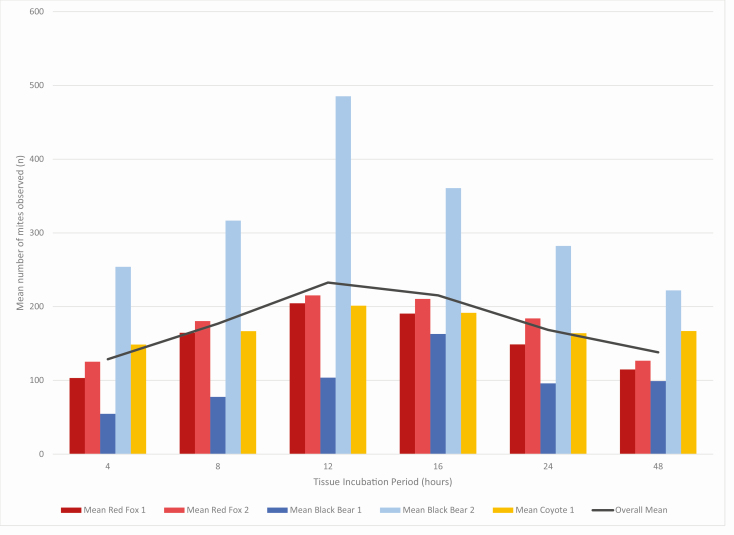
The mean number of mites identified per individual animal from all timing trials conducted on that individual. Mites were counted in each sample at the same six time points—4, 8, 12, 16, 24, and 48 h after sample incubation began in 10% KOH solution at 55ºC. Each color represents a different individual’s mean number of mites identified from all timing trials conducted on tissue from that individual. The black line indicates the overall mean from all mites identified from all high mite burden timing trials conducted, regardless of individual or species.

For each individual animal, the mean number of mites identified at each time point was calculated based on all biopsies from that individual (Fig. 2; Table 1). A consistent pattern was evident with all five individuals. A bell-curve pattern was evident with the fewest mites identified at 4 and 48 h of incubation in digestion solution. The highest mean number of mites were identified after 12 h of incubation for four out of the five individuals, and after 16-h for the fifth individual.

**Table 1.** The mean number of mites identified per individual animal from all high mite burden timing trials conducted on biopsies from that individual

Tissue Incubation (h)	Mean red fox 1	Mean red fox 2	Mean black bear 1	Mean black bear 2	Mean coyote 1	Overall mean
**4**	103	125	55	254	149	129
**8**	165	180	78	317	167	177
**12**	205	215	104	485	201	233
**16**	191	211	163	361	192	215
**24**	149	184	96	282	164	168
**48**	115	127	99	222	167	138

**Fig. 3.**
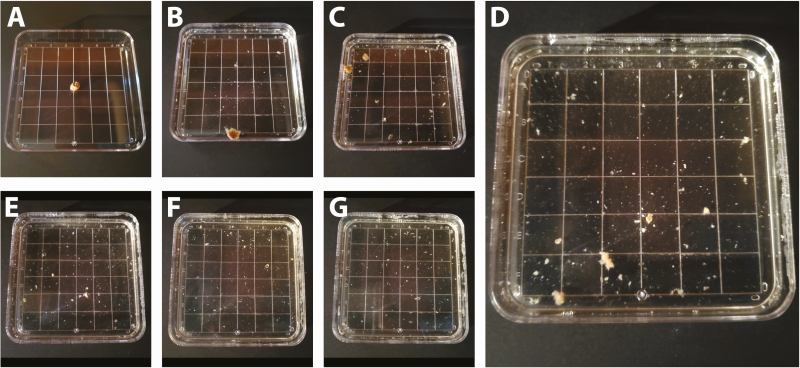
Photographs of Trial P, with tissue from a black bear, at the six specified time intervals used in this study while incubated in 10% KOH at 55°C for a total of 48 h. The time series shows (a) the 6-mm biopsy before the tissue digestion trial began, (b) after 4 h of tissue incubation, (c) after 8 h, (d) after 12 h, (e) after 16 h, (f) after 24 h, and (g) after 48 h of tissue incubation.

At shorter time intervals of 4 and 8 h of tissue incubation, the tissues remained intact with mites still present in tissue tunnels and unable to be counted. This was evident by the larger portions of tissue still present at these time points which obstructed a clear view of the petri dish (Fig. 3b and c), and indicated by the increase in the number of mites identified after 8 h of tissue incubation in nearly all timing trials (Table 2). Additionally, a majority of tissue was dissolved after 12 h of tissue incubation (Fig. 3d). During several trials, mites were more difficult to differentiate from small pieces of tissue after 16 h of incubation (Fig. 3e), compared with being easier to differentiate after 12 h due to a greater difference in size between mites and digested tissue at this time interval. At time intervals of 24 and 48 h of tissue incubation (Fig. 3f and g), only fine fragments of tissue material remained in incubation solution with fewer mites identified at this time point compared with time intervals of 12–16 h of tissue incubation. Mites had often lost their characteristic yellow tint and appeared more translucent or fragmented into pieces and thus, they were more difficult to recognize and count after 24 and 48 h of tissue incubation. The process of tissue degradation was similar across individuals and species (Supp Figs. 1 and 2 [online only]).

**Table 2.** The number of mites identified in each biopsy (‘Trial’) over the six specified time intervals for all high mite burden timing trials conducted

Tissue incubation (h)	Red fox 1	Red fox 1	Red fox 1	Red fox 1	Red fox 1	Red fox 1	Red fox 1	Red fox 1	Red fox 2	Red fox 2	Red fox 2	Black bear 1	Black bear 1	Black bear 1	Black bear 2	Black bear 2	Black bear 2	Coyote 1	Coyote 1	Coyote 1
	Trial A	Trial B	Trial C	Trial D	Trial E	Trial F	Trial G	Trial H	Trial I	Trial K	Trial L	Trial M	Trial N	Trial O	Trial P	Trial Q	Trial R	Trial S	Trial T	Trial U
4	68	78	168	135	166	103	44	64	171	130	75	55	53	56	187	282	293	279	52	115
8	308	138	219	259	100	154	58	80	299	173	69	66	87	80	190	431	329	312	39	149
12	315	202	229	262	226	161	115	126	322	178	146	79	136	96	488	571	397	364	42	198
16	321	205	217	233	174	147	131	97	^*a*^	298	123	133	183	173	302	482	298	357	34	184
24	303	153	150	179	112	103	97	93	252	201	99	65	61	162	297	319	231	307	25	160
48	188	130	123	161	101	82	74	59	126	139	115	48	110	139	173	296	197	257	56	188

^*a*^Data unable to be collected at this time point.

Two individuals with low mite burdens were also evaluated in this study. For the purposes of this study, low mite burden refers to individuals with 10 or fewer mites identified over the course of the digestion timing trials. These tissues came from one black bear and one coyote with signs of mange. Mites were identified from both individuals after 4 h of tissue incubation, and at every subsequent time interval. However, mites were not identified from all replicates of different biopsies from the same individual, with mites identified from two out of four trials conducted on biopsies collected from the coyote and two out of two trials conducted on biopsies collected from the black bear with low mite burden.

### Conclusions

The results of this study highlight the importance of the incubation period to quantifying mite burden in tissue biopsies. The number of mites identified in each individual biopsy varied between each time interval. The results from this study revealed that 12 h of tissue digestion in KOH incubation solution at 55°C resulted in the highest number of mites able to be identified and counted. After 12 h of incubation tissue was dissolved enough to no longer obstruct the view of the petri dish, increasing the observer’s ability to view mites present in the sample. For these reasons, it is recommended to incubate 6-mm tissue biopsies for 12 h before removing the sample and quantifying mite burden.

It should be noted that the number of mites identified during the tissue incubation trials varied, both within each timing trial of an individual biopsy and between the different biopsies from an individual animal. This is particularly noteworthy from the initial trials conducted on the red fox, as these eight trials were all conducted on 6-mm biopsies removed from the same ear of this one individual animal with severe sarcoptic mange. This is important to consider, as even within severely affected lesions there are areas that are more visibly severe, showing thicker skin crusting, higher mite tunneling activity, and often resulting in higher mite densities. These differences in mite burden due to location of sampling should be of high consideration when using tissue biopsies to quantify mite burden, with samples preferentially collected from areas with thickened crusted lesions present. Ideally, several biopsies would be collected and digested to calculate a mean number of mites identified for an individual.

The same procedure outlined in this protocol can be used for the confirmation of *S. scabiei* presence in species known to typically have low mite burdens such as coyotes, or for individuals either known to have low mite burden or in cases when skin scrapes do not confirm presence of mites, with tissue incubated for the same 12-h interval to ensure tissue clearance for best observance of mites present. It is recommended that several biopsies be collected in these cases to better confirm the presence of mites.

Digestion timing was determined to be most critical in striking a balance between removing enough host material to observe all mites present and avoiding over-digesting the sample which resulted in lower mite counts. It is not clear why the number of mites identified decreased when samples were incubated for 24 and 48 h. Mites may remain in solution but be blanched by the KOH solution so that they are no longer observable after this time point, or the KOH may degrade defining features, such as the legs, from prolonged heat and KOH exposure, increasing the difficulty in identifying and counting all mites present.

While there are many studies and applications that use the KOH digestion method, few discuss the specifics of this technique. A wide variation also exists in the timing language that was utilized for this technique. For instance, in a study by Davis and Moon (1990) in which they experimentally infested domestic swine with *S. scabiei* and determined mean mite densities, the authors developed an extraction technique in which pieces of infested skin were placed in 10% hot KOH solution for 5 mins, and then transferred through a filter. While this study had a greater level of detail than many others, the filter used is no longer manufactured and the manufacturer was unable to recommend an adequate replacement. Additionally, since the aim of the present study was to determine mite density in a standardized section of host tissue, a filtration step would risk the loss of mites in the filter itself or with mites still left in the tissue due to short digestion times. Several studies on *S. scabiei* state leaving the digestion solution overnight (i.e., Alasaad et al. 2008, Castro et al. 2016, 2018, Espinosa et al. 2017). While this method likely followed a time-frame similar to the recommended time-frame of 12 h in 10% KOH digestion solution found in this study, leaving a solution overnight allows for variation in digestion time and may result in lower numbers of mites identified than were truly present in the sample.

This protocol provides a detailed procedure for using KOH digestion methods on tissue samples. For studies of *S. scabiei* infestation, this protocol can be used with biopsies from standardized body locations to better determine mange severity, as mange manifests with varied clinical signs dependent on species and individual host response. Studies that utilize different substrates for tissue digestions in solution can also reference this protocol as a starting template for optimizing digestion time and count totals. This is an area of study that warrants further investigation to examine the wide diversity of animals with varying levels of mange severity and mite burden.

## Supplementary Material

ieaa105_suppl_Supplementary_Material
