# Supplementary material for: A Tissue Digestion Protocol for Measuring Sarcoptes scabiei (Astigmata: Sarcoptidae) Density in Skin Biopsies
Source: J Insect Sci. 2020 Nov 2;20(6):20. doi: 10.1093/jisesa/ieaa105 (PMC7604834; doi:10.1093/jisesa/ieaa105)

**Supplemental S1.** Photographs of Trial K, with tissue from a red fox, at the six specified time intervals used in this study while incubated in 10% KOH at 55̊ C for a total of 48 hours. The time series shows a) the 6-mm biopsy before the tissue digestion trial began, b) after 4 hours of tissue incubation, c) after 8 hours, d) after 12 hours, e) after 16 hours, f) after 24 hours, and g) after 48 hours of tissue incubation.


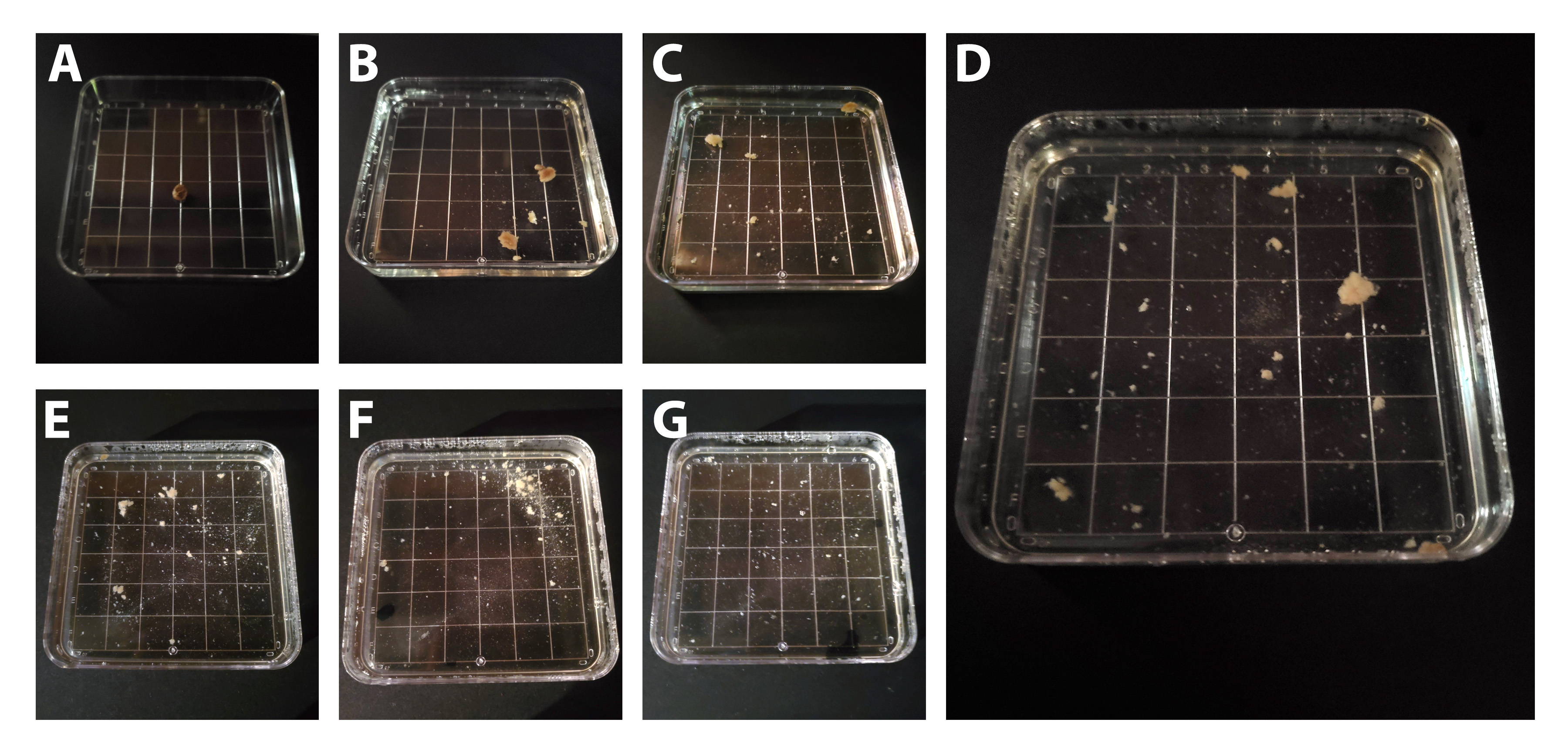


**Supplemental S2.** Photographs of Trial S, with tissue from a coyote, at the six specified time intervals used in this study while incubated in 10% KOH at 55̊ C for a total of 48 hours. The time series shows a) the 6-mm biopsy before the tissue digestion trial began, b) after 4 hours of tissue incubation, c) after 8 hours, d) after 12 hours, e) after 16 hours, f) after 24 hours, and g) after 48 hours of tissue incubation.


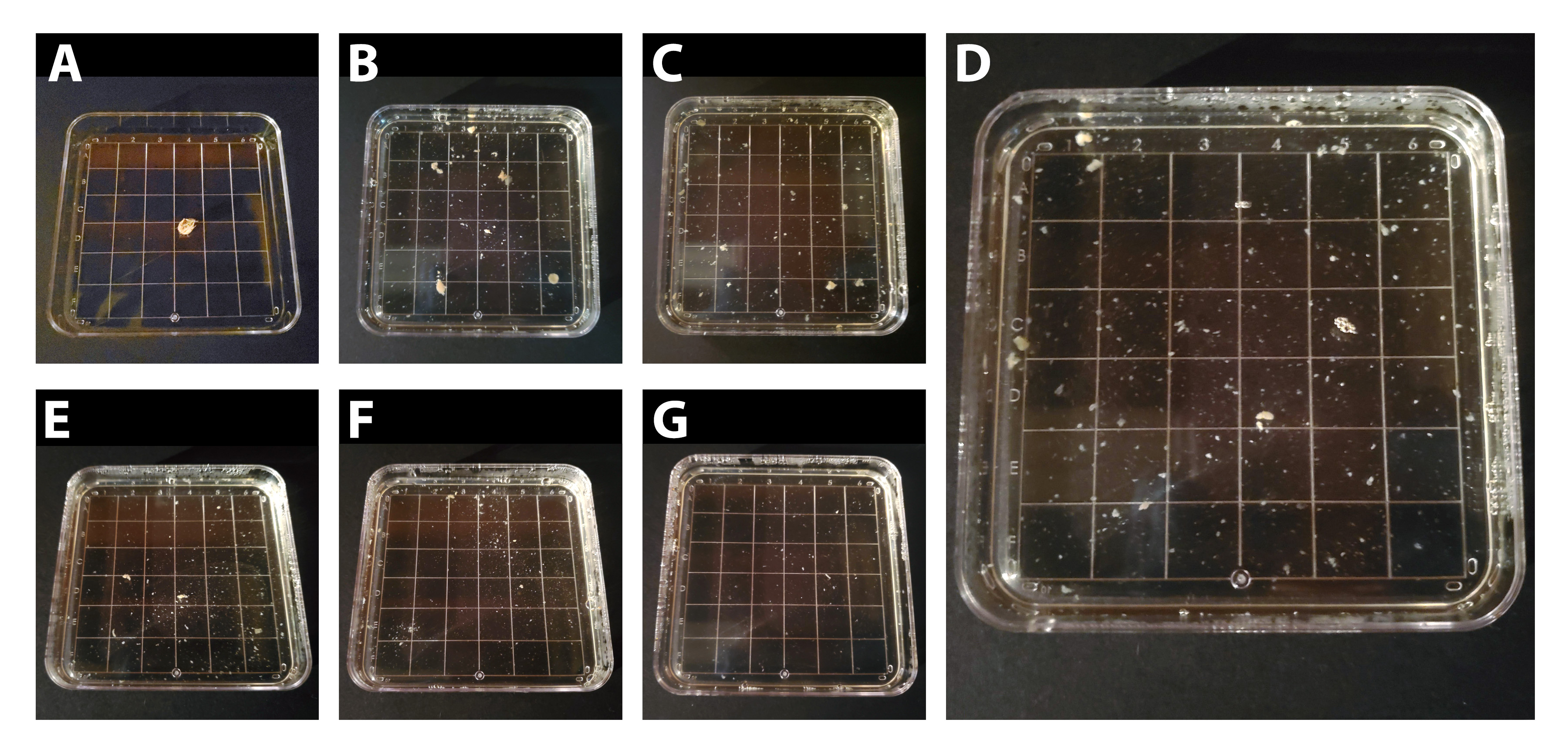

Supplement: ieaa105_suppl_Supplementary_Material [file ieaa105_suppl_supplementary_material.docx]
